# Relationship between Inter-Eye Asymmetries in Corneal Hysteresis and Visual Field Severity in Patients with Primary Open-Angle Glaucoma

**DOI:** 10.3390/jcm12134514

**Published:** 2023-07-06

**Authors:** Tadamichi Akagi, Yukiho Kato-Takano, Daiki Miyamoto, Yuta Sakaue, Ryoko Igarashi, Ryu Iikawa, Mao Arimatsu, Makoto Miyajima, Tetsuya Togano, Takeo Fukuchi

**Affiliations:** Division of Ophthalmology and Visual Science, Niigata University Graduate School of Medical and Dental Sciences, Niigata 951-8510, Japan; tknykh2525@gmail.com (Y.K.-T.); miyadai@med.niigata-u.ac.jp (D.M.); ysakaue@med.niigata-u.ac.jp (Y.S.); iryoko@med.niigata-u.ac.jp (R.I.); ryu-iikawa@med.niigata-u.ac.jp (R.I.); arimatsu@med.niigata-u.ac.jp (M.A.); m-myjm@med.niigata-u.ac.jp (M.M.); ttogano@asahioka-eye.jp (T.T.); tfuku@med.niigata-u.ac.jp (T.F.)

**Keywords:** corneal hysteresis, primary open-angle glaucoma, inter-eye asymmetry, central corneal thickness, corneal resistance factor

## Abstract

This study investigated the influence of asymmetric corneal hysteresis (CH) on asymmetric visual field impairment between right and left eyes in patients with primary open-angle glaucoma (POAG) without a history of intraocular surgery. CH, corneal resistance factor (CRF), and corneal compensated intraocular pressure (IOPcc) were measured using the Ocular Response Analyzer. Differences between the eyes (right eye–left eye: DIF_RL_) and CH-based and in target parameters (higher CH eye–lower CH eye: DIF_CH_) were calculated in the same patient. In 242 phakic eyes of 121 patients, older age (*p* < 0.001), lower CH (*p* = 0.001), and lower CRF (*p* = 0.007) were significantly associated with worse standard automated perimetry (SAP) 24-2 mean deviation (MD). The DIFs_RL_ in axial length (*p* = 0.003), IOPcc (*p* = 0.028), and CH (*p* = 0.001) were significantly associated with the DIF_RL_ in SAP24-2 MD, but not in central corneal thickness (CCT), Goldmann applanation tonometry (GAT) measurement, and CRF. When dividing the patients into two groups based on the median of the CH DIFs_CH_ (0.46), the DIFs_CH_ in CRF (*p* < 0.001), IOPcc (*p* < 0.001), CCT (*p* = 0.004), SAP24-2 MD (*p* < 0.001), and SAP10-2 MD (*p* = 0.010) were significantly different between the groups. Large inter-eye asymmetry in CH is an important explanatory factor for disease worsening in patients with POAG.

## 1. Introduction

Glaucoma is a multifactorial disease. Although elevated intraocular pressure (IOP) is the most important factor associated with glaucoma development and progression [1,2], many other risk factors, including structural characteristics of the cornea and fundus, have been reported to be involved in glaucoma development and progression [3,4,5,6]. In terms of the cornea, not only structural properties, such as thinner central corneal thickness (CCT), but also biomechanical properties, such as lower corneal hysteresis (CH), have been reported to be associated with an individual’s susceptibility to glaucomatous damage [3,4,7,8,9].

Although patients with primary open-angle glaucoma (POAG) usually develop glaucoma binocularly, glaucoma severity is often asymmetric between the two eyes of the same patients. Asymmetric IOP may explain an asymmetric visual field (VF) in many cases [10,11] but not in certain cases [12]. Asymmetric CCT [13] and CH [14] have been suggested to be potential factors associated with the asymmetric severity of glaucoma. However, the type of attention that should be paid to large inter-eye asymmetries in corneal properties in clinical practice is not fully understood.

Two types of corneal biomechanical parameters (CH and corneal resistance factor ([CRF)) and a measure of IOP corrected for these parameters (corneal compensated IOP (IOPcc)) can be measured using the Ocular Response Analyzer (ORA; Reichert, Inc., Depew, NY, USA). In this study, we aimed to investigate the associations of inter-eye asymmetries in CCT, CH, CRF, and IOPcc with inter-eye asymmetries in VF defects in patients with binocular POAG.

## 2. Materials and Methods

This retrospective, observational cross-sectional study was approved by the Research Ethics Committee of Niigata University Hospital and adhered to the tenets of the Declaration of Helsinki.

### 2.1. Participants

The participants were patients with POAG including normal tension glaucoma (NTG), who visited Niigata University Hospital between 1 January 2017 and 31 March 2021. Eyes with NTG were defined when IOP was constantly within normal range (≤21 mmHg) before treatment; eyes with high-tension glaucoma (HTG) were defined when IOP was elevated (>21 mmHg) before treatment. The inclusion criteria were as follows: an open angle on gonioscopy, no history of intraocular surgery including cataract surgery, and no other ocular disease. When either eye did not meet the inclusion criteria, the patient was excluded from this study. 

The patients had undergone a comprehensive ophthalmic examination, including slit-lamp and gonioscopic examinations and measurements of best-corrected visual acuity (using a 5 m Landolt chart), axial length (IOLMaster 500; Carl Zeiss Meditec, Dublin, CA, USA), CCT (CellChek 20; Konan Medical, Nishinomiya, Japan), and CH using the ORA, Goldmann applanation tonometry (GAT), and standard automated perimetry (SAP, Humphrey Visual Field Analyzer; Carl Zeiss Meditec) with the 24-2 and 10-2 Swedish interactive threshold algorithm standard program. The VF results were considered reliable if fixation loss was <15%, the false-positive rate was <15%, and the false-negative rate was <15%.

### 2.2. Measuring CH

CH, CRF, and IOPcc measurements were performed by a trained technician using the ORA. The details of operating this analyzer have been described previously [15,16]. In brief, a continuous jet of air was blown onto the cornea such that it deformed inward forming a slight concavity with increasing air pressure (first applanation point) and subsequently returning (past the second applanation point) to its original position with decreasing air pressure. An optical sensor measured the deflection of the cornea at the first and second applanation points and recorded the air pressure used at each point in the form of two peaks. The CH was the difference between these two applanation pressures. The waveform score reflects the quality of the measurement, and a score of >5 was used as a criterion for inclusion in the analysis. Five measurements were obtained in each eye, and the average of the three middle measurements was considered for analysis. The average waveform score of all the measurements was 7.71 ± 0.81.

### 2.3. Statistical Analyses

The differences between the two eyes of the same patients (DIFs_RL_) were calculated by subtracting the value in the left eye from that in the right eye.

$${DIF}_{RL}=Value\left(right\;eye\right)-Value(left\;eye)$$

To focus on the difference in CH, the CH-based differences (DIFs_CH_) in target parameters between the eyes were calculated by subtracting the values in the eye with lower CH from those in the eye with higher CH in the same patient.

(1) $${DIF}_{CH}=Value\left(eye\;with\;higher\;CH\right)-Value(eye\;with\;lower\;CH)$$

Continuous data were shown as means ± standard deviations (SDs). The Shapiro–Wilk test was performed to assess whether the continuous variables were normally distributed. Non-parametric comparisons of the values were performed using the Mann–Whitney U test, and categorical variables were compared using the chi-square test. The bivariate correlations between factors were examined using Spearman’s rank-correlation coefficient. All statistical analyses were performed using SPSS version 28.0 for Windows (IBM Japan, Tokyo, Japan). *p* < 0.05 was considered statistically significant.

## 3. Results

We included 242 phakic eyes of 121 Japanese patients with POAG (70 men and 51 women) in this study (Table 1). All patients with NTG and HTG had bilateral NTG and HTG, respectively. The mean ± SD of patient age was 60.1 ± 11.6 years. The mean CH, CCT, CRF, IOPcc, and SAP24-2 MD were 8.81 ± 1.34 mmHg, 512.9 ± 38.0 μm, 8.59 ± 1.70 mmHg, 16.4 ± 3.9 mmHg, and −11.12 ± 7.64 dB, respectively. The study included 147 myopic eyes (77 patients) with a refraction of ≤−3 diopters (D), whose mean axial length was 26.20 ± 1.33 mm and mean refraction was −6.4 ± 2.7 D. Sixty-nine eyes (40 patients) had high myopia with a refraction of ≤−8 D, whose mean axial length was 27.05 ± 1.29 mm and mean refraction was −8.5 ± 2.4 D. 

**Figure 1:**
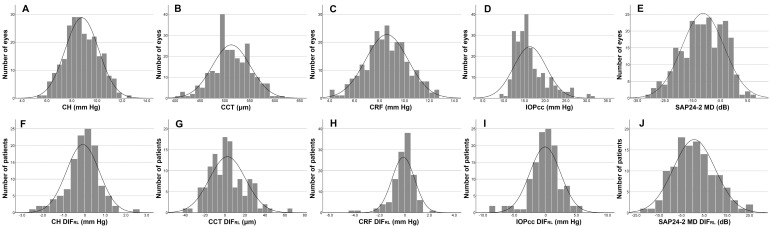
Distribution of Asymmetry in the Analyzed Parameters. (**A**) Corneal hysteresis (CH), (**B**) central corneal thickness (CCT), (**C**) corneal resistance factor (CRF), (**D**) corneal-compensated intraocular pressure (IOPcc), (**E**) standard automated perimetry (SAP) 24-2 mean deviation (MD), (**F**) CH DIF_RL_, (**G**) CCT DIF_RL_, (**H**) CRF DIF_RL_, (**I**) IOPcc DIF_RL_, and (**J**) SAP24-2 MD DIF_RL_. DIF_RL_ = differences between the two eyes (right minus left) of the same patients.

Histograms of the CH, CCT, CRF, IOPcc, and SAP24-2 MD values in all the included eyes are shown in Figure 1A–E. CH, CCT, CRF, and SAP24-2 MD were normally distributed, but IOPcc was not. The differences in the parameters between the two eyes of the same patients (DIFs_RL_) in the CH, CCT, CRF, IOPcc, and SAP24-2 MD values in all the included patients are shown in Figure 1F–J, respectively. Normal distribution was observed in the DIFs_RL_ in CCT and SAP24-2 MD but not in those in CH, CRF, and IOPcc.

Factors associated with SAP24-2 MD were examined in all the included eyes using Spearman’s rank correlation coefficient (Table 2). Older age (*p* < 0.001), lower CH (*p* = 0.001), and lower CRF (*p* = 0.007) were significantly associated with worse SAP24-2 MD. Factors associated with inter-eye asymmetries in SAP24-2 MD were examined in all the included patients (Table 3). The inter-eye asymmetries in axial length (*p* = 0.003), IOPcc (*p* = 0.028), and CH (*p* = 0.001) were significantly associated with inter-eye asymmetry in SAP24-2 MD.

To clarify the characteristics of the patients with large inter-eye asymmetry in CH, the patients were divided into two groups based on the median of the DIFs_CH_ in CH (0.46): 61 and 60 patients were included in the small and large CH DIF_CH_ groups, respectively (Table 4). There were no significant differences in age and sex between the groups. The DIFs_CH_ in CRF (*p* < 0.001), IOPcc (*p* < 0.001), CCT (*p* = 0.004), 24-2 MD (*p* < 0.001), and 10-2 MD (*p* = 0.010) were significantly different between the groups, which indicates that CRF and CCT tend to be smaller, IOPcc tends to be larger, and visual field tends to be worse in the eyes with lower CH in the large CH DIF_CH_ group. The DIFs_CH_ in GAT IOP and axial length were not significantly different between the groups.

## 4. Discussion

In the current study, the inter-eye asymmetry in CH was significantly associated with that in the severity of VF loss, but the inter-eye asymmetry in CRF or CCT was not. The results also suggest that large bilateral difference in CH (>0.46 in this study) is of significant importance in understanding a bilateral difference in VF disorder in POAG patients. 

CH and CRF are considered a measure of ocular viscous properties and measure of the overall ocular rigidity, respectively, and they are thought to be affected by several ocular conditions. It was reported that CH was positively associated with CCT and was negatively associated with age, IOP, and axial length [17]. In the current study, CH was significantly lower in eyes with HTG than in those with NTG. A previous study showed that the mean CH and IOPcc were significantly higher, and the mean CRF was significantly lower in NTG than in HTG, which is consistent with our results [18]. High IOP was suggested to affect corneal biomechanical properties. On the other hand, Kaushik et al. [16] examined patients who had not received ophthalmic treatment and reported that CH was lower in eyes with POAG and NTG than in healthy eyes, but CH was not lower in eyes with ocular hypertension (OHT). Laiquzzaman et al. [19] reported that CH measurements were almost constant throughout the day and were not associated with the diurnal IOP variation. These results indicate that increased IOP alone cannot explain the low CH in eyes with POAG and that increased viscoelasticity of ocular tissue may have a protective role against glaucoma [20]. 

Few reports have investigated the differences in CH between the two eyes of the same subject. It was reported that a worse VF was associated with lower CH, but not with CCT, in POAG patients with an asymmetric VF [14,21]. Lower CH was also reported to be associated with glaucoma progression, but CCT was not [4,7,22,23], and Estrela et al. [24] showed that a difference in CH between the eyes was significantly associated with asymmetric rates of VF progression. Glaucoma severity differed between their report (mean SAP24-2 MD: better eyes, −4.19 dB and worse eyes, −3.98 dB) [24] and ours (mean SAP24-2 MD: total eyes, −11.12 dB), which indicates that asymmetric CH may be more associated with asymmetric glaucoma progression than different glaucoma onset.

The reason for the close association of lower CH with worsening glaucoma has not yet been clarified. It has been indicated that the biomechanical characteristics of the cornea may be associated with the viscoelastic properties of the lamina cribrosa, posterior sclera, and other optic nerve head (ONH) structures [8,25]. CH was reported to be associated with deeper or more posteriorly curved lamina cribrosa negatively and with the reversal of lamina cribrosa displacement following IOP reduction positively [25,26,27,28]. In this study, because the association between ONH structures and CH was not examined, it is still uncertain how the ONH structures differed between the eyes of patients in the large CH DIF_CH_ group. Other studies have shown that systemic oxidative stress potentially influenced the glaucomatous damage and affected CH or CRF in older female glaucoma patients [29,30]. Because both eyes in the same patient should be affected by systemic oxidative stress to the same degree, it is quite unlikely that our results are associated with systemic oxidative stress. However, attention should be paid to the fact that CH could be affected by various factors. Further studies investigating various factors, including ONH structures, as well as corneal biomechanical properties, would be helpful in revealing the mechanism underlying the association of CH with worsening glaucoma. 

In the current study, eyes with more myopia tended to show less VF loss in the same patients (Table 3). There appears to be a consensus on the effects of myopia on the risk of glaucoma development [31]. However, whether or not myopia is a risk factor for glaucoma progression has been a topic of controversy. While a study reported that severe myopia might be a significant risk factor for VF progression [32], others showed that myopia might be a protective factor against VF progression [33,34]. On performing an inter-eye comparison, Lee et al. [35] reported that the more myopic eye in a myopic NTG patient exhibited more severe VF loss; this finding was not consistent with our result, but the evaluation methods of myopia were different between their study (assessed by refractive error) and ours (assessed by axial length). CH was reported to be negatively associated with axial length in control eyes but not in eyes with glaucoma [17]. Because glaucoma is a multifactorial disease, various factors could affect the inter-eye asymmetry in VF loss. A two-group comparison based on the CH DIF_CH_ showed significant associations with the DIFs_CH_ in SAP24-2 MD and SAP10-2 MD. Consequently, it can at least be said that a large difference in CH (e.g., ≥0.46 in this study) between the eyes of the same patient is a significant factor that could explain the asymmetry in VF loss in this patient even though the association between myopia and glaucoma progression could not be clarified in the current study. 

In conclusion, CH is an important factor involved in glaucoma progression. A large difference in CH should be considered an important explanatory factor for disease worsening in POAG patients. 

## Figures and Tables

**Table 1:** Demographic and ophthalmic characteristics of the patients.

	Total		NTG	HTG	*p* Value *
Characteristics	Values	Range	Values	Values	
By eye	(N = 242)		(N = 116)	(N = 126)	
Axial length (mm)	25.35 (25.35) ± 1.63	21.74–30.03	25.35 (25.30) ± 1.66	25.34 (25.35) ± 1.61	0.94
CCT (μm)	512.9 (511) ± 38.0	401–612	516.8 (512.5) ± 38.1	509.2 (509.5) ± 37.7	0.21
GAT (mmHg)	14.1 (14) ± 3.7	7–28	12.6 (12.5) ± 2.6	15.5 (15) ± 4.0	<0.001
Glaucoma medications (n)	2.8 (3) ± 1.2	0–5	2.6 (3) ± 1.2	2.9 (3) ± 1.2	0.035
CH (mmHg)	8.81 (8.67) ± 1.34	5.55–12.64	9.04 (9.02) ± 1.29	8.59 (8.46) ± 1.36	0.012
CRF (mmHg)	8.59 (8.62) ± 1.70	4.24–12.62	8.33 (8.10) ± 1.66	8.83 (8.78) ± 1.70	0.014
IOPcc (mmHg)	16.4 (15.5) ± 3.9	9.2–31.4	14.8 (14.4) ± 2.8	17.8 (16.4) ± 4.3	<0.001
SAP24-2 MD (dB)	−11.12 (−10.70) ± 7.64	−30.25 to 6.36	−10.95 (−11.01) ± 7.05	−11.27 (−10.46) ± 8.17	0.89
SAP10-2 MD (dB)	−10.77 (−10.19) ± 7.90	−34.23 to 1.78	−10.12 (−9.45) ± 7.42	−11.38 (−10.82) ± 8.31	0.30
By subject	(N = 121)		(N = 58)	(N = 63)	
Age (yrs)	60.1 (63) ± 11.6	19–82	58.8 (62) ± 11.3	61.3 (63) ± 11.9	0.32
Sex (male/female)	70/51	-	32/26	38/25	0.57
AL Abs-DIF	0.24 (0.14) ± 0.46	0.00–4.51	0.27 (0.16) ± 0.60	0.21 (0.13) ± 0.30	0.32
CCT Abs-DIF	14.01 (11) ± 11.65	0–65	13.14 (10) ± 11.40	14.81 (14) ± 11.91	0.38
GAT Abs-DIF	1.0 (1.0) ± 1.3	0–8	0.9 (1) ± 1.1	1.1 (1) ± 1.5	0.68
Glaucoma medications Abs-DIF	0.2 (0.0) ± 0.6	0–4	0.2 (0) ± 0.7	0.2 (0) ± 0.5	1.0
CH Abs-DIF	0.58 (0.46) ± 0.55	0.00–2.59	0.52 (0.35) ± 0.56	0.63 (0.49) ± 0.53	0.073
CRF Abs-DIF	0.65 (0.44) ± 0.65	0.04–4.10	0.57 (0.44) ± 0.45	0.73 (0.44) ± 0.78	0.51
IOPcc Abs-DIF	1.81 (1.42) ± 1.67	0.05–8.81	1.57 (1.23) ± 1.44	2.03 (1.54) ± 1.83	0.18
24-2 Abs-DIF	7.30 (6.39) ± 5.60	0.00–26.41	7.07 (6.34) ± 5.20	7.52 (6.39) ± 5.97	0.84
10-2 Abs-DIF	7.88 (6.34) ± 6.27	0.01–29.46	7.12 (5.61) ± 5.67	8.59 (7.00) ± 6.76	0.29

Data (apart from those related to sex) are presented as the mean (median) ± standard deviation. Abs-DIF = absolute difference between the eyes in the same patient; CCT = central corneal thickness; CH = corneal hysteresis; dB = decibels; CRF = corneal resistance factor; GAT = Goldmann applanation tonometry; HTG = high-tension glaucoma; IOPcc = corneal-compensated intraocular pressure; NTG = normal-tension glaucoma; SAP = standard automated perimetry. * Comparison between NTG and HTG was performed using the Mann–Whitney U test, except for sex (the chi-square test).

**Table 2:** Factors associated with SAP24-2 MD (dB) in total eyes.

	ρ	*p* Value *
Age (yrs)	−0.302	<0.001
Axial length (mm)	0.013	0.84
CCT (μm)	0.087	0.18
GAT (mmHg)	0.028	0.67
IOPcc (mmHg)	−0.050	0.44
CH (mmHg)	0.204	0.001
CRF (mmHg)	0.173	0.007

CCT = central corneal thickness; CH = corneal hysteresis; CRF = corneal resistance factor; dB = decibels; GAT = Goldmann applanation tonometry; IOPcc = corneal-compensated intraocular pressure; MD = mean deviation; SAP = standard automated perimetry. * Bivariate correlations with SAP24-2 MD are investigated using Spearman’s rank correlation coefficient.

**Table 3:** Bivariate correlations between inter-eye asymmetries in SAP24-2 MD (dB) and other variables.

	ρ	*p* Value *
Axial length DIF_RL_ (mm)	0.272	0.003
CCT DIF_RL_ (μm)	0.080	0.38
GAT DIF_RL_ (mmHg)	−0.158	0.084
IOPcc DIF_RL_ (mmHg)	−0.199	0.028
CH DIF_RL_ (mmHg)	0.291	0.001
CRF DIF_RL_ (mmHg)	0.126	0.17

CCT = central corneal thickness; CH = corneal hysteresis; CRF = corneal resistance factor; dB = decibels; DIF_RL_ = difference between the two eyes of the same patients was calculated by subtracting the value in the left eye from that in the right eye; GAT = Goldmann applanation tonometry; IOPcc = corneal-compensated intraocular pressure; MD = mean deviation; SAP = standard automated perimetry. * Bivariate correlations with inter-eye asymmetries in SAP24-2 MD are investigated using Spearman’s rank correlation coefficient.

**Table 4:** Comparison between patients with small inter-eye asymmetries and those with large inter-eye asymmetries in CH.

	Small CH DIF_CH_ (≤Median) (N = 61)	Large CH DIF_CH_ (>Median) (N = 60)	
	Median (25th Percentile, 75th Percentile)	Median (25th Percentile, 75th Percentile)	*p* Value *
Age (yrs)	62 (51, 66)	63 (54,68)	0.20
Sex (male/female)	36/25	34/26	0.79
CH DIF_CH_ (mmHg)	0.19 (0.06, 0.30)	0.78 (0.59, 1.21)	-
CRF DIF_CH_ (mmHg)	0.18 (−0.14, 0.47)	0.54 (0.19, 1.09)	<0.001
GAT DIF_CH_ (mmHg)	0 (0, 1)	0 (−1, 0)	0.075
IOPcc DIF_CH_ (mmHg)	−0.18 (−1.04, 0.69)	−1.53 (−3.14, −0.55)	<0.001
Axial length DIF_CH_ (mm)	0.00 (−0.16, 0.14)	0.01 (−0.12, 0.12)	0.86
CCT DIF_CH_ (μm)	0.0 (−10.0, 6.0)	8.5 (−5.0, 22.5)	0.004
SAP24-2 MD DIF_CH_ (dB)	−0.46 (−7.65, 3.74)	4.77 (−0.08, 9.56)	<0.001
SAP10-2 MD DIF_CH_ (dB)	−2.64 (−6.64, 3.31)	3.15 (−3.14, 8.57)	0.010

CCT = central corneal thickness; CH = corneal hysteresis; CRF = corneal resistance factor; dB = decibels; DIF_CH_ = CH-based difference calculated as the value of the target parameter in the eye with higher CH minus that in the eye with lower CH in the same patient; GAT = Goldmann applanation tonometry; IOPcc = corneal-compensated intraocular pressure; SAP = standard automated perimetry. * Mann–Whitney U test for continuous variables and chi-square test for categorical variables.

## Data Availability

The data analyzed in this study are available from the corresponding author on reasonable request.
